# A Comparative Analysis of Butter, Ghee, and Margarine and Its Implications for Healthier Fat and Oil Group Choices: SWOT Analysis

**DOI:** 10.1002/fsn3.4557

**Published:** 2024-10-29

**Authors:** F. Mohammadi‐Nasrabadi, A. Rashidimehr, Kh. Khoshtinat, B. Alhouei, A. Massomian, M. Rashidian, F. Esfarjani

**Affiliations:** ^1^ Food and Nutrition Policy and Planning Research Department, Faculty of Nutrition Sciences and Food Technology National Nutrition and Food Technology Research Institute, Shahid Beheshti University of Medical Sciences Tehran Iran; ^2^ Department of Microbiology and Food Hygiene, Faculty of Veterinary Medicine Lorestan University Khorramabad Iran; ^3^ Department of Food Science and Technology, Faculty of Nutrition Sciences and Food Technology, National Nutrition and Food Technology Research Institute Shahid Beheshti University of Medical Sciences Tehran Iran; ^4^ Department of Food Science and Technology, Science and Research Branch Islamic Azad University Tehran Iran

**Keywords:** butter, fatty acid profile, gas chromatography, ghee, margarine, SWOT analysis

## Abstract

This study aims to comparatively analyze butter, ghee, and margarine fatty acid profiles and their implications for healthier fat and oil group choices. In this cross‐sectional study, 60 samples from best‐selling brands of butter, ghee, and margarine were randomly selected from five food chain stores in Tehran, Iran. Then, all the samples were coded, packed in cool conditions, and sent to the laboratory to determine the fatty acid profiles by using gas chromatography (GC). Based on the authors' experiences and the available literature, a policy dialogue session was held with stakeholders about oil and fat challenges, followed by a Strengths, Weaknesses, Opportunities, and Threats (SWOT) analysis. The mean ± SE of total fatty acids in butter, ghee, and margarine was 94.07 ± 0.17, 94.49 ± 0.61, and 99.00 ± 0.18; total saturated fatty acid (TSFA) 66.69 ± 0.39, 64.26 ± 0.63, and 40.36 ± 0.87; trans fatty acid (TFA) 2.43 ± 0.09, 3.60 ± 0.29, and 0.83 ± 0.15 g/100 gfat, respectively. The predominant TFAs in butter and ghee were *vaccenic* acid (animal source) (2.06 ± 0.07 and 2.41 ± 0.17), while in margarine, it was *elaidic* acid (plant‐based source) (0.32 ± 0.12 g/100 gfat), respectively. Also, the SWOT findings showed being TFAs in the acceptable range (as the main strength), mismanagement (Weakness), reducing taxes based on lower TFA content (Opportunity), and sanctions (as the main Threat) were the most important criteria affecting fat choices in the Iranian food basket. The results of the study found that butter, ghee, and margarine contain relatively low levels of TFAs; however, butter and ghee were rich in beneficial fatty acids, which have been shown to have health‐protective properties. Policymakers can implicate the proposed strategies and opportunities from the SWOT analysis for healthier fat and oil choices to promote public health.

## Graphical abstract

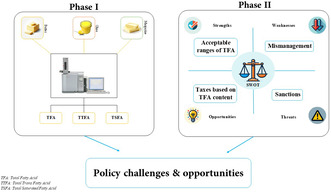

## 1 Introduction

Butter and margarine are lipid‐based substances predominantly in food basket (Ahmad and Ahsan 2020; Cerone and Smith 2021; Yun et al. 2020). The optimal food basket is a scientific and powerful tool for developing food and nutrition policies, executive planning, and performing evaluation of programs to ensure food security and maintain nutritional public health (Caputo and Lusk 2022).

Nonetheless, in the contemporary era, margarine has surpassed butter in market competition (Lampe and Sharp 2014). However, margarine possesses a higher proportion of trans fatty acids (TFAs) and a greater degree of unsaturation compared to butter, primarily resulting in margarine's heightened susceptibility to oxidation (Weber et al. 2022). The butter and margarine markets are experiencing significant changes driven by health trends, consumer preferences for natural products, and economic factors. While butter is gaining market share due to its health perception and culinary versatility, margarine is facing challenges that may hinder its growth. Ghee continues to thrive in specific markets, particularly in South Asia, as it aligns with the demand for healthier cooking fats (Rashidi and Shabani 2017). The combined market for these products reflects a growing trend toward both traditional dairy and plant‐based options, catering to diverse consumer preferences (Beyene 2019; Idahe and Zemedu 2017).

Milk‐fat products like butter, butter‐oil blends, fractionated butter fats, and ghee are processed and sold globally. Butter generally contains over 80% lipid, and various types of butter are found in the worldwide market. In the United States, butter oil refers to the fat extracted from butter. Ghee has around 99.6% milk fat and less than 0.1% moisture. The key distinctions between butter oil and ghee are as follows: (1) ghee is made at temperatures ranging from 100°C to 140°C, while butter oil is produced by melting butter between 60°C and 80°C and (2) ghee has lower moisture and different fatty acids and phospholipids compared to butter oil (Ramadan 2023).

Ghee is a dairy product that possesses lipophilic properties and is frequently employed as a medium for culinary purposes. It is derived from the milk of cows or sheep. Ghee is also made from buffalo as well as goat milk and possesses a pleasing scent (Bali and Utaal 2019; Pajohi‐Alamoti, Kahledian, and Bazargani‐Gilani 2019; Pena‐Serna and Restrepo‐Betancur 2020). The advantages of butter and ghee stem from their possession of conjugated linoleic acid (CLA), which contributes to their antioxidative and antiatherogenic characteristics (Sindhuja et al. 2020). Ghee is also an important carrier of fat‐soluble vitamins (A, D, E, K) and essential fatty acids (linolenic acid and arachidonic acid), apart from having rich and pleasant sensory properties, and is rich in vitamin K2 and CLA (Kumar et al. 2018).

CLA is a term used for positional and geometric isomers of linoleic acid that can be found in animal fats such as beef, lamb, and dairy foods. Grass‐fed beef may have higher levels of CLA than grain‐fed beef. CLA is produced by gut microbial including some species of potential probiotic *bifidobacteria* from fermentaion of PUFAs and isomerization of linoleic acid in the rumens of ruminant's animal food products. CLA is essentially a type of polyunsaturated fat. In other words, it is technically a trans fat—but a natural type of trans fat that occurs in many healthy foods (Sindhuja et al. 2020).

The World Health Organization (WHO) attempted to reduce the daily intake of TFAs as well as emphasized the controlled intake of saturated fats in response to the proven negative effects of trans isomers and the rise in the incidence of NCDs. In 2003, Denmark became the first nation to adopt a 2% trans restriction policy. Then, this strategy was adopted by other nations, such as Switzerland (2008), Austria (2009), Island (2011), Hungary (2014), and Norway (2014) (World Health Organization 2015). The European Food and Nutrition Action Plan (2015–2020) has also put forth a plan to reduce TFA consumption to less than 1% of daily caloric intake (Costa et al. 2016). Having a particular goal of a 25% reduction by 2025, Iran is one of the few nations having a national action plan for the prevention and management of NCDs. Various initiatives have been carried out across the nation to reach the objective (Amini, Doustmohammadian, and Ranjbar 2021). A 23% increase in cardiovascular risk has been linked to a 2% absolute increase in energy intake (EI) from trans fat (Remig et al. 2010). Margarine, biscuits, french fries, cereal‐based meals, fast food, snacks, milk, baked goods, pies, and cakes are the primary sources of industrial TFAs in the area which have been linked to a range of health hazards (Dhaka et al. 2011). Traditional foods have been found to have lower TFA contents (Jawaldeh and Al‐Jawaldeh 2018). iTFAs are thought to be responsible for about 500,000 fatalities annually and raise the risk of heart disease (World Health Organization 2021). The WHO recommends that the intake of TFAs not exceed 1% of total daily EI or less than 2.2 g per day in a diet of 2000 cal (Hoteit et al. 2021).

The reduction of total fat consumption, saturated fatty acids (SFAs), and dietary cholesterol has been a fundamental aspect of dietary recommendations in previous years. However, there has been a recent shift toward prioritizing the quality of dietary fat intake, the elimination of iTFA, and the replacement of SFA with MUFAs and PUFAs (Schwingshackl et al. 2022).

To our knowledge, this is the first study to determine the fatty acids profiles of butter, ghee, and margarine and highlight the Strengths, Weaknesses, Opportunities, and Threats (SWOT) analysis to identify practical solutions for the development of industry and to facilitate policymakers' decisions to implicate healthier fat and oil choices to promote public health.

## 2 Materials and Methods

### 2.1 Phase I

In this cross‐sectional study, 60 samples from best‐selling brands of butter, ghee, and margarine were randomly selected from food chain stores in five districts (north, south, west, east, and center) of Tehran, Iran (2024). The list of the most purchased samples was taken from the marketing unit of the Iranian Fats & Oil Association.

Then, all samples were coded with a two‐digit number and packed in cool conditions to transport the items to the laboratory for analysis.

Samples were quickly chilled at 4°C (ghee) and −18°C (butter and margarine) before being prepared at the lab. Before the test got started, it was stored at the room temperature. Research has demonstrated that the exposure of ghee to elevated temperatures leads to a significant increase in free fatty acids, peroxides, and TBA levels. Furthermore, a notable association with the duration of storage at high temperatures has been observed, and the effect of storage temperature on the development of peroxides and TBA of ghee samples was significantly greater than the effect of the storage period. The refrigeration of butter oil has been identified as an effective means of preserving its inherent properties (Asha et al. 2015; Darwish 2024).

#### 2.1.1 Reagents and Standards

Analytical grade solvents and reagents were used, including methanol, n‐hexane, sodium hydroxide, methanolic boron trifluoride, and sodium chloride, all purchased from Merck (Darmstadt, Germany). The certified standard fatty acid methyl ester (FAME) mixture of 37 components, obtained from Supelco (Bellefonte, PA, USA), was used for identification purposes.

##### 2.1.1.1 Sample Preparation

###### 2.1.1.1.1 Fat Extraction

To extract butterfat before methylation, the process typically involves several steps:

1. Cream separation: Start by separating cream from milk. This can be done using a cream separator, which utilizes centrifugal force to separate the lighter cream from the heavier milk.
2. Churning: The separated cream is then churned to produce butter. During churning, the fat globules coalesce and form butter, while buttermilk is expelled.
3. Washing: The butter may be washed with cold water to remove residual buttermilk and impurities, which helps in obtaining a purer fat.
4. Melting: The butter is then melted gently to convert it into a liquid form. This process can be done using a water bath or a low heat source to avoid overheating.
5. Filtration: After melting, the liquid butter is filtered to remove any remaining solids or impurities, resulting in a clear butterfat.
6. Storage: The extracted butterfat can be stored in a suitable container, typically under refrigeration, until it is ready for methylation.

###### 2.1.1.1.2 To Extract Margarine Before Methylation

Melting the Margarine

Step 1: Begin by gently heating the margarine to melt it. This can be done in a water bath or on a stove at low heat to avoid degradation of the fat.
Step 2: Once melted, allow the margarine to cool slightly but not solidify.

Separation of Fat

Step 3: Pour the melted margarine into a separatory funnel or a centrifuge. If using a blender, blend the melted margarine to ensure uniformity.
Step 4: If using a separatory funnel, allow the mixture to settle. The fat will rise to the top, while water and other components will settle at the bottom.
Step 5: Carefully drain the aqueous layer from the bottom, leaving the fat layer intact. If using a centrifuge, spin the mixture to separate the fat from the other components effectively.
Step 6: The extracted fat may still contain impurities. To purify, you can filter the fat through a fine mesh or cheesecloth to remove any solid residues.
Step 7: Optionally, the fat can be washed with warm water to remove any remaining impurities. After washing, allow it to settle again and separate the water.
Step 8: The extracted fat should be dried to remove any residual moisture. This can be done by gently heating it again or using a vacuum desiccator.

###### 2.1.1.1.3 Extraction of Ghee Fat

Heating and Simmering

- Butter is heated in a pan or pot over medium heat until it melts completely.
- The melted butter is then simmered, allowing the water content to evaporate and the milk solids to settle at the bottom of the pan.

Skimming and Filtering

- The clear, golden liquid on top is the ghee, which is carefully skimmed off using a spoon or ladle.
- The ghee is then filtered through a fine mesh strainer or cheesecloth to remove any remaining milk solids or impurities.

Cooling and Storage

- The filtered ghee is allowed to cool to room temperature (Hewavitharana et al. 2020).

##### 2.1.1.2 Methylation

Methyl‐esterification of samples used in the analyses was performed by BF3‐MeOH method according to AOCS (Delmonte et al. 2009). In a 125‐mL flask, 1 g of oil was weighed and mixed with 10 mL of 0.5 (N) methanolic sodium hydroxide solution. The mixture was heated at 100°C for 10 min until the fat was fully dissolved. Next, 12 mL of BF3‐MeOH reagent was added, and the mixture was heated for an additional 2 min. After cooling to room temperature, 5 mL of n‐hexane was added. The reaction was stopped by adding 15 mL of a saturated solution of NaCl and shaking the flask for about 15 s. The tube was then left to settle until two layers are formed. The upper organic phase, which consisted of 1 mL of supernatant, was transferred to a tube containing 1 g of anhydrous material. The mixture was then filtered through a 0.22‐μm disposable syringe filter before being transferred to gas chromatography (GC) vials and injected into the GC. Some advantages of using GC are high resolution, high accuracy, high speed, high sensitivity, and repeatability (Schreiner 2005).

### 2.2 GC Analysis for Fatty Acid Determination

The GC–Flame Ionization Detector (Dahlan et al. 2020) method was used to profile fatty acids. Triheneicosanoin was combined with the extracted fat, and the resulting mixture underwent methylation to produce FAMEs. Following GC separation, the FAMEs were identified using the pertinent standards. Analysis indicated the grams of fatty acid per 100 g of the food sample and the amount as the % of the total fatty acids. A description of the utilized equipment and oven program, along with the mass spectrometry (MS) approach that was chosen, are provided below.

A 2‐mL microcentrifuge tube was filled with 15 μL of the extracted fat portion (15 μL) from the butter and margarine or butter‐oil samples, 1.5 mL of hexane, and 15 μL of 2 mol/L potassium hydroxide in methanol. During phase separation, the mixture was vortexed for 1 min and then put into a vial holder for 45 seconds. After being transferred into a second microcentrifuge tube with 0.2 g of anhydrous sodium sulfate, the upper phase (hexane portion, 1 mL) was rotated at 16,753× *g* for 5 min. Before being prepared for GC analysis, the top phase of the solution (0.75 mL) was carefully put into a glass GC vial and kept at 18°C. An Agilent 6890 series (Santa Clara, CA, USA) GC with a flame ionization detector (Dahlan et al. 2020), an autosampler (HP G1513A), and a tray were used to examine the prepared samples. An HP‐88 column (100 m × 0.25 mm × 0.2 μm) (Agilent, Santa Clara, CA, USA) was used to separate the fatty acids. The GC system was adjusted to 250°C, and 1 μL of material with a 120:1 split ratio was injected. The oven was set up as follows: it was held at 120°C for 1 min, then increased to 175°C in increments of 10°C; held again for 10 min, then increased to 210°C (5°C/min); held again for 5 min, then increased to 230°C (5°C/min); and held again for 2 min. The temperature selected for the detector was 280°C.

For every sample, a duplicate GC analysis was performed (Salas‐Valerio et al. 2022). Each sample weighed 100 g and included the following components: total lipids, unsaturated fatty acids (USFs), SFAs, and TFAs. SFA plus TFA added together equals total fat. The FA content attributed to the sum of 19 isomers (C4:0 (Bu), C6:0 (Co), C8:0 (Cy), C10:0 (Ci), C12:0 (L), C14:0(M), C14:1 (MO), C16:0 (P), C16:1 (P‐O), C18:0 (S), TC18:1 (T.O), C18:1 (O), TC18:2 (T.LE), C18:2 (LE), C18:3 (Munisekhar et al. 2022), TC18:3 (TLN), C20:0 (Ar), C20:1 (Ga), and C22:0 (B)).

### 2.3 Phase II

#### 2.3.1 The SWOT Analysis

The starting point will always be a SWOT analysis, which is an instrument used to identify strengths, weaknesses, opportunities, and threats. This analysis helps to devise an approach to analyze the situation and develop new strategies (Stolovitch and Keeps 2006). The SWOT analysis is a method for creating strategic options based on a situational analysis. The SWOT matrix is a useful tool for managers and policymakers to develop strategies and achieve specific goals. It provides a framework for identifying and formulating strategies. The tool is effective and efficient, allowing for the observation of possible upcoming changes through a systematic approach to introspection (Dahlan et al. 2020).

A policy discussion session was organized to verify the strategies that had been obtained in the earlier stages and to assess the viability and potential roadblocks of the strategies to build a policy. Participants from various stakeholder groups gathered for a policy debate to focus on a topic that interests them all, though it need not be a shared. This approach can be a useful source of data and answers for policymakers since it assumes that people in various circumstances and settings have varied perspectives on an issue. The specific objectives of policy dialogues might vary based on the time of the dialogue and the level of the policy creation process, even though their primary objective is to support informed policy decision making (Robert et al. 2020).

Therefore, the meeting was held with the presence of different groups of people with different backgrounds, expertise, and views in the field of oils and fats in order to gather a set of different views. The samples were selected by a purposive sampling method. First, the consent of the participants to attend the policy discussion meeting was obtained through phone or email. Due to the existence of conflict between different groups of producers, the meeting was held at the National Nutrition and Food Research Institute as a neutral institution.

At the beginning of the policy discussion session, after introducing the attendees, and taking into account the timing of the meeting, the research team presented a summary of the research conducted on butter, ghee, and margarine in fat choices of Iranian food baskets and the challenges and factors affecting it. Then, the participants were asked to give their opinions regarding the priority obtained among the strategies, weaknesses or limitations of each group of strategies, feasibility, and possible obstacles, according to the purpose of the study and the strategies obtained from the study (SURE Collaboration 2011; Toorang et al. 2022). The authors conducted the SWOT analysis based on their experiences and also a policy dialogue session on the relevant literature on the subject. Additionally, quantitative results were utilized to enhance the SWOT analysis.

In the present study, using the SWOT technique, the potentials and limitations of the region were identified, and practical solutions were proposed for the development of policy strategies to optimize the food choices of butter, ghee, and margarine in food baskets to promote public health.

### 2.4 Statistical Analysis

All examinations for each sample were performed three times. Data analysis was conducted using IBM's Statistical Package for Social Sciences (SPSS) software, version 21. The data were summarized using counts and percentages for categorical variables and mean (standard error) for quantitative variables. To test the differences between butter and margarine, a one‐way analysis of variance (ANOVA) followed by Tukey's test was used. The limit for TFA content in 100 g of fat is 2 g. Items that exceed this limit were considered to have an elevated TFA composition (World Health Organization 2019). *p* values less than 0.05 were considered significant.

## 3 Results and Discussion

### 3.1 The Fatty Acid Composition of Butter, Ghee, and Margarine

In butter and ghee, the main SFAs were *palmitic* (30.21 ± 0.20, 30.94 ± 0.66), *stearic* (8.10 ± 0.12, 9.56 ± 0.32), and *myristic acid* (11.28 ± 0.20, 11.10 ± 0.19 g/100 g), respectively. Whereas in margarine, the dominant SFAs were *palmitic* (31.56 ± 1.15) and *stearic acid* (7.90 ± 1.48). The primary USF in butter and ghee was *oleic acid* (18.22 ± 0.24 and 21.12 ± 0.70), whereas in margarine, they were *oleic acid* (36.98 ± 1.81) and *linoleic acids* (19.67 ± 1.25). The predominant TFAs in butter and ghee were *vaccenic acid* (2.06 ± 0.07 and 2.41 ± 0.17), while in margarine, it was *elaidic* acid (0.32 ± 0.12), respectively (Table 1). The *vaccenic* acid, which is the TFA found in butter and ghee, is synthesized by the microflora in the rumen of cows.

**TABLE 1:** Fatty acid composition of butter, ghee, and margarine (g/100 g).

Fatty acids	Butter (*n* = 24)	Ghee (sheep milk) (*n* = 12)	Standard (%)^a^, ^b^	Margarine (*n* = 24)	Standard (%)^c^
	(mean ± SE)			(mean ± SE)	
*Saturated fatty acids*					
C4:0 (Bu)	4.14 ± 0.16	2.60 ± 0.36	1–4	ND	—
C6:0 (Co)	3.12 ± 0.07	2.15 ± 0.26	0.8–3	ND	—
C8:0 (Cy)	1.91 ± 0.05	1.33 ± 0.14	0.5–1.7	ND	—
C10:0 (Ci)	3.95 ± 0.11	2.89 ± 0.23	1.7–3.9	ND	—
C12:0 (L)	3.96 ± 0.11	3.69 ± 0.17	2.3–4.5	0.17 ± 0.02	≤ 2
C14:0 (M)	11.28 ± 0.20	11.10 ± 0.19	5.4–14.5	0.74 ± 0.04	—
C16:0 (P)	30.21 ± 0.20	30.94 ± 0.66	25–41	31.56 ± 1.15	—
C18:0 (S)	8.10 ± 0.12	9.56 ± 0.32	6–15	7.90 ± 1.48	—
C20:0 (Ar)	0.31 ± 0.00	0.66 ± 0.09	—	0.00 ± 0.00	—
C22:0 (B)	0.15 ± 0.00	0.16 ± 0.02	—	0.14 ± 0.02	—
*Monounsaturated fatty acids*					
C14:1 (MO)	1.15 ± 0.01	1.15 ± 0.04	0.5–1.7	ND	—
C16:1 (P‐O)	1.69 ± 0.01	1.75 ± 0.11	1–6	ND	—
TC18:1 (T.O)	2.06 ± 0.07	2.41 ± 0.17	1.06–3.48	0.32 ± 0.12	—
C18:1 (O)	18.22 ± 0.24	21.12 ± 0.70	18–33.4	36.98 ± 1.81	—
C20:1 (Ga)	0.18 ± 0.08	0.11 ± 0.00	—	0.10 ± 0.04	—
*Polyunsaturated fatty acids*					
TC18:2 (T.LE)	0.37 ± 0.03	0.56 ± 0.07	0.32–1.15	0.41 ± 0.06	—
C18:2 (LE)	2.97 ± 0.08	2.48 ± 0.18	0.9–3.7	19.67 ± 1.25	≥ 15
C18:3 (LN)	0.28 ± 0.00	0.32 ± 0.03	0.8–3.3	0.90 ± 0.20	—
TC18:3 (TLN)	ND	ND	—	0.10 ± 0.04	—
TSFA	66.69 ± 0.39	64.26 ± 0.63	—	40.36 ± 0.87	≤ 48
Total trans	2.43 ± 0.09	3.60 ± 0.29	—	0.83 ± 0.15	≤ 2
Total	94.07 ± 0.17	94.49 ± 0.61	—	99.00 ± 0.18	—

*Note:* Each value is the mean ± SE (standard error) from three independent experiments.

Abbreviations: Ar, arachidic acid; B, behenic acid; Bu, butyric acid; Ci, capric acid; Co, caproic acid; Cy, caprylic acid; Ga, gadoleic acid; L, lauric acid; LE, linoleic acid; LN, linolenic acid; M, myristic acid; MO, myristoleic acid; ND, not detected; O, oleic acid; P, palmitic acid; P‐O, palmitoleic acid; S, stearic acid; T.LE, trans‐isomer of linoleic acid; TLN, trans‐isomer of linolenic acid; T.O, trans‐isomers of oleic acid (vaccenic from animal source and eladic acid from plant‐based source); TSFA, total of saturated fatty acid.

^a^ Iran National Standardization Organization 162.

^b^ Iran National Standardization Organization 1254.

^c^ Iran National Standardization Organization 143.

The composition of fatty acids in butter and ghee is influenced by various factors such as age the breed of the animal, its diet, the season, genetic variations, and the period of lactation (Esmaeili Fard, Bahmaei, and Eshratabadi 2016). Fatty acid composition of milk may vary due to reasons such as the type of the milk, genetic and physiological factors of animals, lactation, season, feed, and geographic location. Therefore, the fatty acid content of butter, like other dairy products, is highly affected by these factors (Çetinkaya 2021). Butter contains butyric acid at about 4% of total fatty acids (Lindmark Månsson 2008). Furthermore, it has been found that the concentration of butyric acid in milk fat (3%–4%) is higher than that in all other types of natural fats (Ramadan 2023). Also, short‐ and medium‐chain fatty acids are synthesized in the mammary glands of ruminants, so regional differences may be due to differences in mammary cell regulation between different breeds and genotypes of cattle (Tian et al. 2023).

The study results showed that the sampled butter and ghee had average levels of SFA at 66.69 and 64.26, MUFA at 21.06 and 24.02, and PUFA at 3.25 and 2.8, respectively.

**FIGURE 1:**
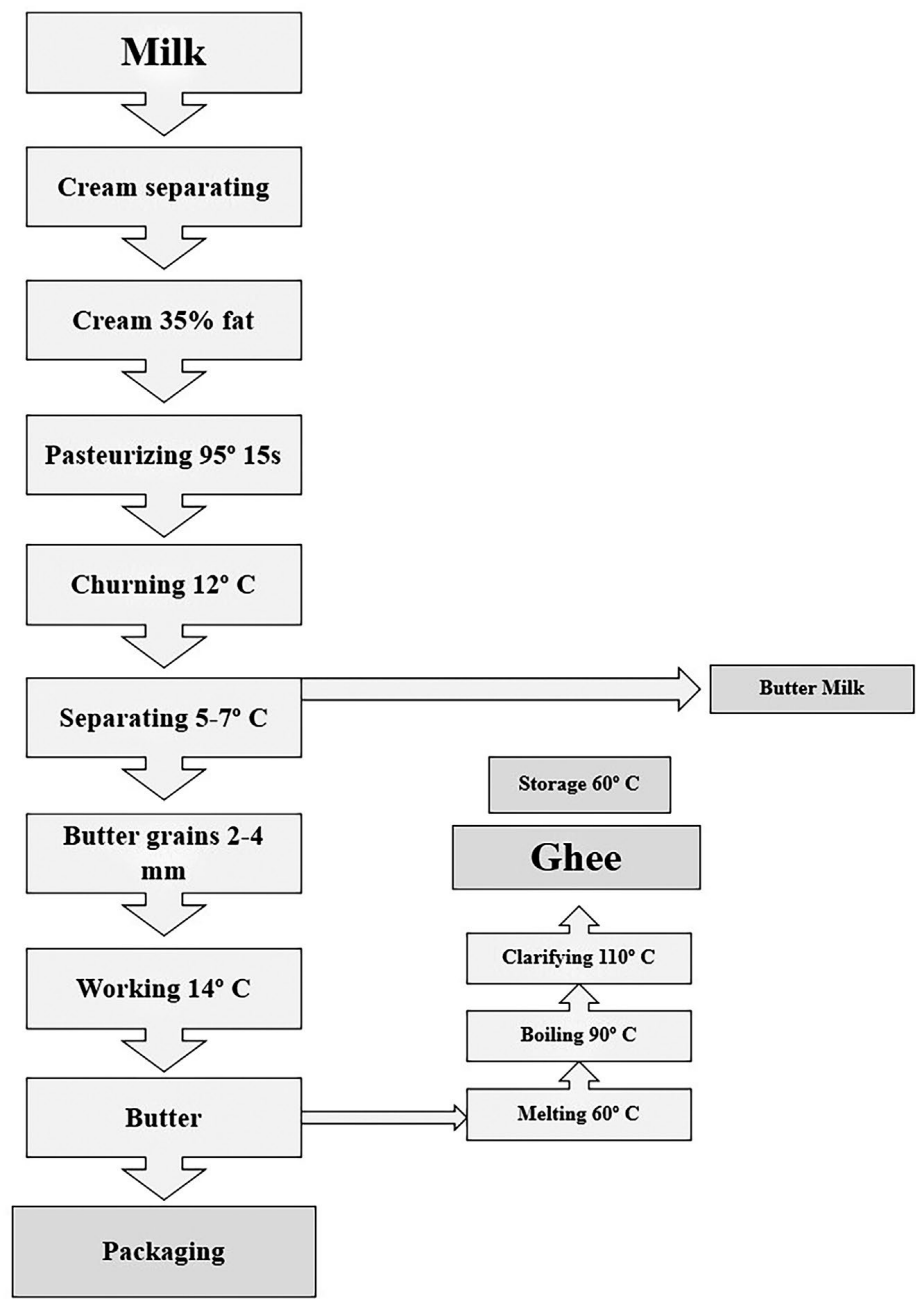
The preparation process of ghee.

Ghee is manufactured using traditional techniques in rural regions. To prepare this particular type of ghee, the initial step involves fermenting the milk (in this case, sheep milk) to generate yogurt, which is then left at room temperature for one night. Subsequently, the yogurt is subjected to agitation within a customary circular churning apparatus for a few hours, thereby facilitating the separation of butter fat from the yogurt. Following the melting of this butter fat and the subsequent elimination of any impurities, the remaining fat is referred to as ghee (Figure 1).

Studies showed that the fatty acid composition of butter and ghee has varying impacts on NCDs, including neurodegenerative illnesses and cardiovascular disease (CVD). Research has indicated that TFA generated from butter may accelerate the development of dyslipidemia and atherosclerosis (AS), which may ultimately cause CVD to worsen (Khodadadi, Heshmati, and Karami 2017; Munisekhar et al. 2022; Sharma et al. 2018; Wei et al. 2022). Conversely, eating butter that is abundant in CLA has been linked to neutral or maybe positive effects on lipid profile. CLA, a natural trans fat present in ghee, may also decrease serum LDL levels (M. Kumar et al. 1999). Moreover, studies have shown that consuming ghee can have significant hypolipidemic and hypocholesterolemic effects. The hypocholesterolemic effect of ghee may be attributed to the increased excretion of bile constituents. Cholesterol and its metabolites are primarily excreted in bile (Kumar, Sambaiah, and Lokesh 2000). The study found that dietary ghee increases the excretion of various substances in bile, including cholesterol, phospholipids, bile solids, uronic acid, total bile acids, taurocholic acid, and ursodeoxycholic acids. The results suggest that ghee may have an impact on bile composition (M. V. Kumar, Sambaiah, and Lokesh 2000).

Furthermore, it has been suggested that using particular lipids present in butter and ghee—specifically CLA—can help prevent or treat neurological disorders like dementia, amyotrophic lateral sclerosis (ALS), Parkinson disease, and Huntington disease which are characterized by an energy deficit (Roy et al. 2007). In peripheral tissues, CLA incorporation is prompt and its deposition occurs particularly in neutral lipids (NLs). CLA and its desaturated and elongated metabolites are likely biosynthesized and then transported to extrahepatic tissues, as evidenced by their high concentration also in plasma and adipose tissue after dietary CLA administration. Given that modification of FA profile in the brain by dietary FAs is quite difficult, this is also true for dietary CLA. In rats fed with a diet supplemented with 150 mg/day of 9c,11t or 9t,11t or 10t,12c or 10t,12t isomers for 6 days, only minor changes in CLA brain concentrations were found. This might be due to either (1) a preferential incorporation of CLA in tissue TAG and the relative virtual lack of this lipid fraction in brain tissue or (2) a very selective and steady incorporation of FAs in the brain and a rapid CLA metabolism to other conjugated FAs in the brain. Fa and co‐workers in 2005 administered a single dose of CLA (2 g by gavage) to female Sprague–Dawley rats to monitor its incorporation and metabolization up to 24 h. Confirming previous research, CLA incorporation was much lower in the brain than that in the other tissues examined. At 24 h, CLA isomer concentrations were both increased by four‐folds in plasma and liver and two‐folds in brain, whereas in adipose tissue, 9c,11t isomer increased six‐folds and t10,c12 by four‐folds. However, a relatively high accumulation of CLA metabolites was found, particularly products of peroxisomal β‐oxidation related to the content of the precursor. The discrete levels of the two CLA isomers measured in plasma could be ascribed to the different rates of hydrolyzation of the isomers in chylomicron TAG by lipoprotein lipase. In the brain, the level of the t10,c12 isomer was lower than that of the c9,t11 isomer probably because of the enhanced metabolism of t10,c12 for c9,t11, as shown by higher concentrations of t10,c12 metabolites. t10,c12 seemed to be β‐oxidized very efficiently in all tissues, particularly in the brain. Products of peroxisomal β‐oxidation of CLA were detected in experiments in vivo and in vitro, confirming that CLA could act as a ligand to brain PPARα. Future studies are needed to investigate whether dietary CLA may exert anti‐inflammatory activity, particularly in the setting of neurodegenerative diseases and neuropsychiatric disorders with a neuroinflammatory basis. Future studies are needed to investigate whether dietary CLA may exert anti‐inflammatory activity, particularly in the setting of neurodegenerative diseases and neuropsychiatric disorders with a neuroinflammatory basis (Tian et al. 2023).

Overall, the effects of butter's fatty acid composition on various NCDs can vary, which emphasizes how crucial it is to comprehend the precise lipid components and how they affect the onset and course of disease (Munisekhar et al. 2022; Meena 2013; Roy et al. 2007).

A meta‐analysis conducted on nine studies across 15 countries has determined that butter exhibits a neutral or feeble association with overall mortality, CVD, and diabetes (Pimpin et al. 2016).

The results showed a total mean SFA:40.36, MUFA:36.98, and PUFA:20.57 g/100 g in margarine. The majority of them were in the standard range (Table 1).

**FIGURE 2:**
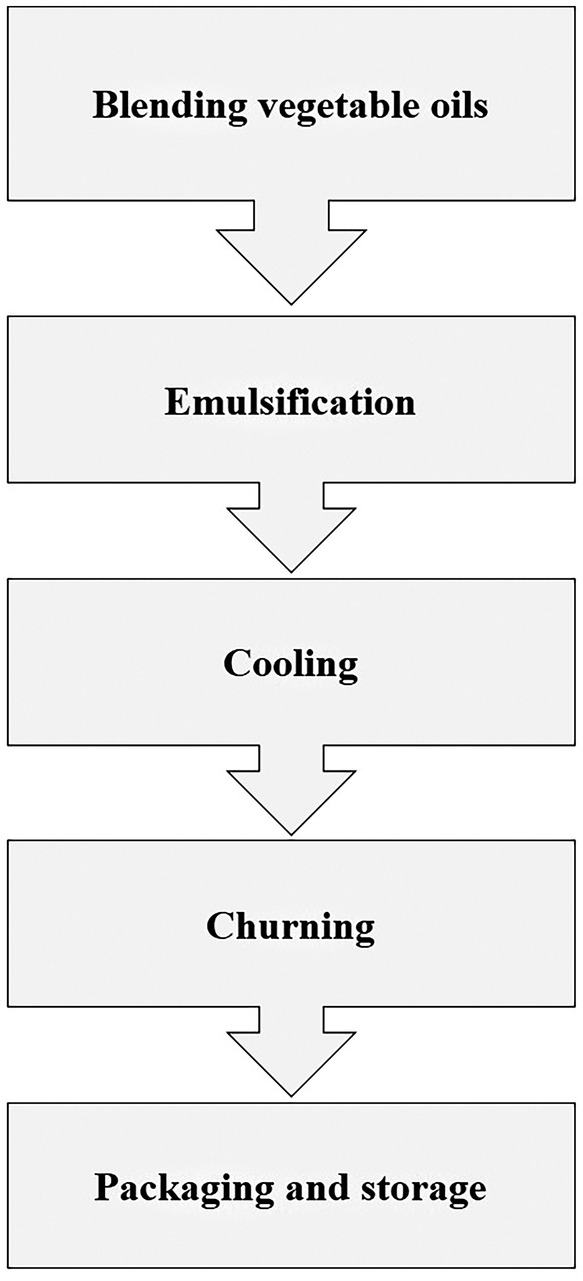
The preparation process of margarine.

Margarine is a water‐in‐oil emulsion that consists of a minimum of 80% fat and a maximum of 16% water. In comparison to butter, margarine contains a higher amount of TFAs and unsaturation due to its susceptibility to oxidation. In the past, nonselective hydrogenation was utilized in the production of margarine, leading to the formation of products with elevated levels of SFAs and unfavorable rheological properties (Figure 2). Trans fat is present in margarine made from vegetable oils that have undergone industrial partial hydrogenation. The primary source of TFAs in the human diet is semi‐hydrogenated oils (PHOs), which can contain 10%–60% trans fat. In Korea, TFAs range from 2% to 7%. Studies indicate that margarine made with PHOs contains about 40% trans fat and minimal lauric and myristic acid, while margarines made without PHOs have negligible trans fat content (Salas‐Valerio et al. 2022).

The predominant fatty acid found in such products was stearic acid, which contributes to their firm texture (Esmaeili Fard, Bahmaei, and Eshratabadi 2016). The outcome of an investigation conducted in 2016 indicated that butter outperformed margarine in terms of qualitative aspects. Nevertheless, if the hydrogenation procedure employed in the production of margarine is eliminated and substituted with alternative secure techniques, it can result in products of elevated nutritional worth (Esmaeili Fard, Bahmaei, and Eshratabadi 2016). The fatty acid profile composition of margarine typically depends on the vegetable oil utilized. In general, the margarines studied showed higher levels of USFs compared to SFAs, with MUFA content surpassing PUFA levels. This aligns with the variety of ingredients like canola, soy, sunflower, corn, coconut, and palm kernel used in their production. These results may have variations in the plant due to differences in climate, soil, season, water, harvest time, transportation, and other variables (Abramovič et al. 2018).

### 3.2 Comparison of TFA Content in Butter, Ghee, and Margarine

**FIGURE 3:**
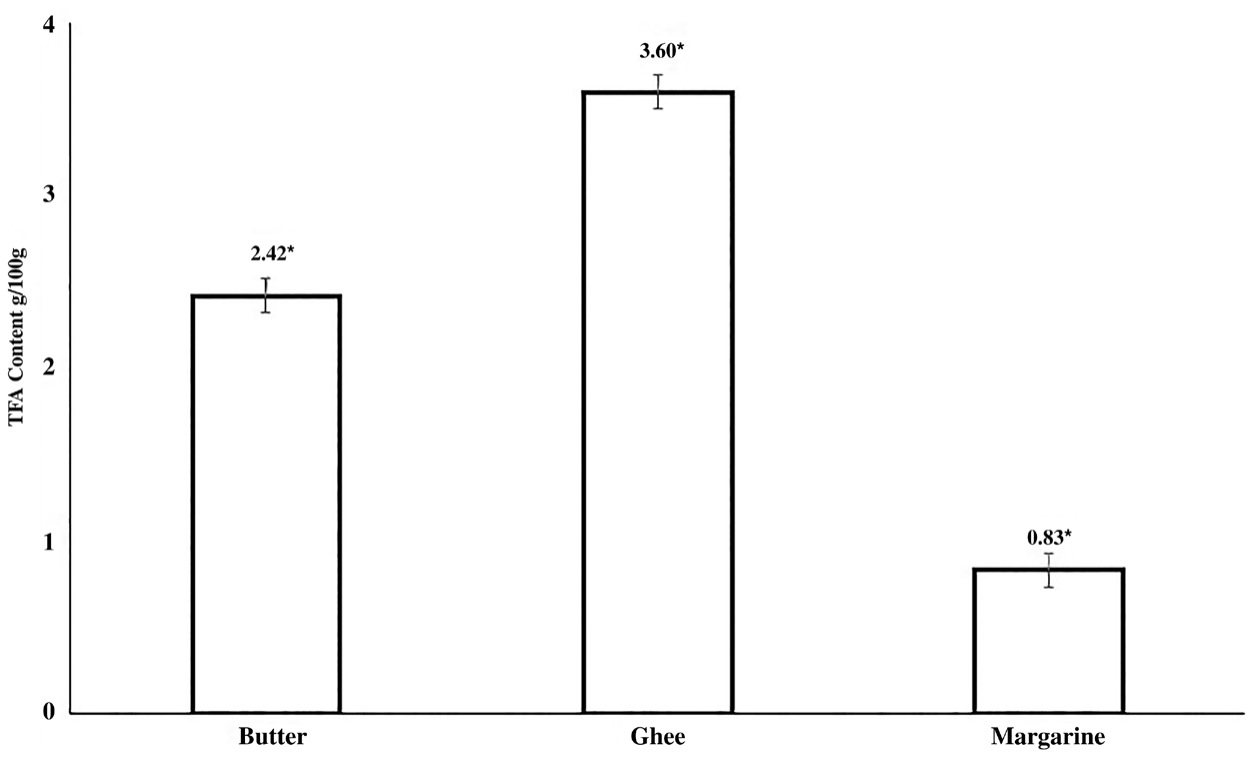
The comparison of TFA content in butter, ghee, and margarine.

The results showed that mean ± SE of TFAs in butter (2.43 ± 0.09), ghee (3.60 ± 0.29), and margarine (0.83 ± 0.15) was close to the acceptable ranges (≤ 2 for butter and margarine and ≤ 5 for ghee) (Tarar et al. 2020). The comparison between the mean values of TFAs in butter, ghee, and margarine with a one‐way ANOVA test showed significant differences (*p* = < 0.001) (Figure 3). The TFA consumption from ruminants does not seem to pose a health risk. A study found a negative correlation between the incidence of cancer and rTFA intake (Samet‐Bali, Ayadi, and Attia 2009). Studies revealed no discernible correlation between the risk of NCDs and rTFAs (Ali et al. 2016; Munisekhar et al. 2022; Meena 2013; Wendeu‐Foyet et al. 2023). These results raise the possibility that industrially manufactured TFAs, as opposed to those found naturally in ruminant meat and milk, are the source of the harmful health effects associated with TFAs. It is significant to note that most research examined how industrial TFAs affected health outcomes, suggesting that there may be a higher risk associated with them (Mohammadi et al. 2023; Niforou et al. 2022). It is important to emphasize that evidence derived from both epidemiological studies and preclinical experimental models consistently supports the notion that TFA derived from ruminant fat, consumed at typical levels, has either a neutral or advantageous impact on health (Gebauer et al. 2011).

Margarine can serve as a viable substitute for butter, particularly due to its high saturation level. Moreover, the production of products containing CLA and the adjustment of the omega‐6 to omega‐3 ratio can effectively address the deficiency of these compounds in the diet (Esmaeili Fard, Bahmaei, and Eshratabadi 2016).

Another study compared the effects of consuming dairy fat containing rTFA and margarine containing iTFA. The results showed that the different types of TFAs present in butter and margarine had significantly different effects on blood lipid profiles and metabolome profiles, which are indicators of CVD risk (Guggisberg et al. 2022). Overall, butter and margarine have been studied for their impact on CVD. One study found that the consumption of margarine, which contains iTFA, was specifically associated with inflammation, CVD, and type 2 diabetes (Guggisberg et al. 2022). Therefore, more research is needed to determine the relative impact of butter, ghee, and margarine on CVD.

Research has examined the relationship between NCDs such as type‐2 diabetes and CVDs and the fatty acid composition of margarine. Research findings indicate that margarine did not exhibit statistically significant impacts on levels of glucose, triglycerides, cholesterol, or both when compared to butter (Tarar et al. 2020). Dietary TFAs from margarine have been shown to raise LDL and total cholesterol levels, which are coronary heart disease risk factors. Also, consumption of iTFA from margarine has been particularly linked to NCDs (type 2 diabetes, CVD, and inflammation). To completely comprehend the impact of margarine fatty acid content on NCDs, more research is necessary as the impacts are complex. Another study compared the effects of consuming dairy fat containing rTFA and margarine containing iTFA. The results showed that the different types of TFAs present in butter and margarine had significantly different effects on blood lipid profiles and metabolome profiles, which are indicators of CVD risk (Guggisberg et al. 2022).

In a recent scoping review, it summarized comprehensively total fat intake as 25%–35% (≤ 30%), SFA 7%–10%, MUFA ≤ 10%, PUFA ≤ 10%, and TFA 1%–2% (Schwingshackl et al. 2021; Shankar et al. 2005). Available published evidence makes it reasonable to recommend replacing SFA with MUFA and PUFA and avoiding the consumption of industrial TFA. In addition, it should be noted that not only replacement is important but also the source of food containing MUFA and PUFA replaced is of particular importance (Hosseinabadi and Nasrollahzadeh 2022; Hosseini and Asgary 2012; Mohammadifard et al. 2010; Shankar et al. 2005).

The consideration of various types of fatty acids and the range of foods containing SFAs should be included in the WHO guidelines on saturated fat, with a particular emphasis on their potential effects on health outcomes (Astrup et al. 2019).

Recently, the allowed amount of TFA intake has been under debate. Mozaffarian et al. suggested that “the intake of trans fatty acids (TFA) from commercially available, partially hydrogenated vegetable oils exhibits an elevated propensity toward the development of risk factors associated with coronary heart disease (CHD)” (Liu et al. 2017; Mozaffarian, Aro, and Willett 2009; Mozaffarian and Clarke 2009).

Based on the American Heart Association (AHA) and American Diabetes Association (ADA), butter and ghee are sources of saturated fat, which can increase the risk of CVD. It is recommended to limit the intake of SFA to less than 10% of total daily calories (Lichtenstein et al. 2021; Liu et al. 2017).

### 3.3 The Achievements in Eliminating Industrial TFAs

Eliminating industrial TFAs from foods has significant health benefits. In 2004, Denmark became the first country in the world to restrict industrially produced TFAs in all food products (Ghebreyesus and Frieden 2018). Pakistan is one of the countries that has a high intake of TFAs as the main risk factor for noncommunicable diseases. Efforts are being made in this country to eliminate industrially produced TFAs in the food supply chain in line with the priority goals of the WHO. It was advised to advocate for initiatives aimed at substituting conventional ghee with more nutritious options; establish and execute optimal regulatory procedures consistent with guidelines from the WHO; and revise regulations on food labeling to ensure the provision of transparent details to guide consumers in making healthy dietary decisions (Tarar et al. 2020). Until 2007, there was no limit on the amount of SFAs and TFAs in semi‐hydrogenated oil in Iran's policy‐making authorities. This year to improve the quality of edible oils in line with the nutritional health of society, the limit of SFAs and TFAs (maximum amount of 20% and 30%, respectively) was made mandatory in the formulation of the national standard “Household Oil.” The policy of reducing the number of TFAs in household oil is gradual, and in 2011, this amount reached less than 10%, and until now, this trend is decreasing. In the past two decades, many measures have been taken to change the composition of edible oil consumption in order to improve the health of the Iranian community (Esmaeili et al. 2021).

More than 100 countries have yet to take action to reduce iTFA from their national food supplies. Fifty‐eight countries have passed laws that protect more than 3 billion people from TFA complications yet (World Health Organization 2021). The US Food and Drug Administration (FDA) has declared that partially hydrogenated oils (PHOs) are no longer generally recognized as safe. This decision prohibits the use of PHO in food products, effectively banning trans fats in the US food supply (US FDA 2018). Partial hydrogenation, a process that involves adding hydrogen to oils, is commonly used in products like margarine to produce solid fats at room temperature. However, during the partial hydrogenation process, TFAs are formed. In food products containing PHO and TFAs, it is advisable to limit the use of hard or sticky margarine, as well as reduce the intake of fats and oils high in SFAs and cholesterol (Weber et al. 2022).

### 3.4 SWOT Analysis of Butter, Ghee, and Margarine

Findings from the SWOT analysis showed that reformulations, milk quality, equipment, packaging, transportation, and public literacy can guide policymakers and improve food industries to optimize the food choices of butter, ghee, and margarine in food baskets to promote public health (Tables 2 and 3).

**TABLE 2:** The SWOT matrix to optimize butter, ghee, and margarine in Iranian food basket fat choices: policy strengths, weaknesses, opportunities and threats.

Internal factors	
Strengths	Weaknesses
Acceptable range of TFAs in products Multilateral expertise and technical knowledge The educability of consumers	Mismanagement Old equipment and packaging Lack of knowledge about butter, ghee, and margarine among consumers Low quality of ruminants' feeding Lack of tracking from farm to factory

External factors	
Opportunities	Threats
Reducing taxes by lowering TFA content Training and education from farmer to consumer Reformulation of food products	Sanctions Seducing advertisement Currency fluctuations

**TABLE 3:** Strategies for the development of butter, ghee, and margarine in Iranian food basket fat choices.

Invasive strategies (SO)	Conservative strategies (WO)
A thorough inspection from farm to consumer (WHO guidelines) Increasing public awareness through campaigns	Using eco‐friendly and modern technologies A healthy diet and food safety training from mass media

Competitive strategies (ST)	Defensive strategies (WT)
More interaction between the government and the oil and fat industry Increasing knowledge about product safety	Supporting feed production by modern technology domestically Strengthen public information infrastructure on food safety

Abbreviations: SO, strength–opportunity; ST, strategy–threat; WO, weakness–opportunity; WT, weakness–threat.

### 3.5 Recommended Actions for Policymakers and Food Industry

#### 3.5.1 Policymakers

- The policymaker should plan and act on how the quality and quantity of ruminant feed to increase the levels of CLA.
- Implement regulations to reduce TFAs in fat products, as well as possible.
- Promote awareness of the differences between butter, oil, and margarine, including composition, health effects, and use in cooking.
- Promote resources such as food labeling and nutritional education campaigns.
- Policymakers should collaborate with healthcare professionals and nutritionists.
- Promote media literacy.

#### 3.5.2 Food Industry

- Substitution of TFAs and SFAs with monounsaturated and polyunsaturated fats to produce “healthier” margarine.
- Interesterification, the adjunction of high‐melting glycerides, and the joint effect of both have also been explored to modify the solid fat content in ghee.
- Improving the quality of the factory including safety, hygiene, and up‐to‐date equipment.
- Training and education of dairy farmers and workers on milk safety and hygiene practices.
- Implementing sustainable packaging materials and practices.
- Conducting consumer research and gathering feedback.
- Implementing traceability from farm to factory

## 4 Future Research

In order to assist consumers in identifying and selecting foods that are appropriate sources of healthy fats, future dietary recommendations should concentrate on healthy dietary patterns. Furthermore, to successfully communicate evidence‐based scientific information to the public, dietary advice must take into account and incorporate principles for effective scientific communication as a high priority (Liu et al. 2017).

Nutrition scientists are reliable subject matter experts (Fiske and Dupree 2014). Therefore, for consumers to make wise, evidence‐based dietary decisions, it is crucial that they successfully communicate study findings to policymakers, authoritative bodies, and the general public. Furthermore, sharing scientific discoveries has been proposed as a component of official academic instruction and might be seen as a civic obligation (Brownell, Price, and Steinman 2013).

### 4.1 Limitations

The content of CLA in butter and ghee can be challenging to accurately quantify due to limitations in GC techniques used in laboratories. The lack of a standard guide for butter, ghee and margarine at the national (Iran) and international levels was accounted as an important limitation of the study.

## 5 Conclusion

The results of the study found that butter, ghee, and margarine in Iran contain relatively low levels of TFAs. However, butter and ghee are rich in beneficial fatty acids, which have been shown to have health‐protective properties. Therefore, when consumed in moderation, they may offer health benefits due to their favorable fatty acid composition. The SWOT analysis findings showed that TFAs were in the acceptable range (S), mismanagement (W), reducing taxes based on lower TFA content (O), and sanctions (T) were the most important criteria affecting optimized choice in the food basket. Policymakers can implicate the proposed strategies and opportunities from the SWOT analysis for healthier fat and oil choices to promote public health.

## Author Contributions

**F. Mohammadi‐Nasrabadi:** data curation (equal), formal analysis (equal), methodology (equal), validation (equal), writing – original draft (equal), writing – review and editing (equal). **A. Rashidimehr:** conceptualization (equal), methodology (equal), writing – original draft (equal), writing – review and editing (equal). **Kh. Khoshtinat:** formal analysis (equal), methodology (equal), resources (equal), validation (equal), writing – original draft (equal), writing – review and editing (equal). **B. Alhouei:** conceptualization (equal), investigation (equal), methodology (equal), resources (equal), visualization (equal), writing – original draft (equal), writing – review and editing (equal). **A. Massomian:** conceptualization (equal), data curation (equal), methodology (equal), resources (equal), writing – original draft (equal), writing – review and editing (equal). **M. Rashidian:** data curation (equal), investigation (equal), methodology (equal), project administration (equal). **F. Esfarjani:** conceptualization (equal), data curation (equal), formal analysis (equal), funding acquisition (equal), investigation (equal), methodology (equal), project administration (equal), resources (equal), software (equal), supervision (equal), validation (equal), visualization (equal), writing – original draft (equal), writing – review and editing (equal).

## Ethics Statement

The authors have nothing to report.

## Conflicts of Interest

The authors declare no conflicts of interest.

## Data Availability

The data will be available upon request from the corresponding author.
